# P-2294. Listeriosis in cancer patients: Uncommon and protean

**DOI:** 10.1093/ofid/ofae631.2447

**Published:** 2025-01-29

**Authors:** Robin Snellings, Takahiro Matsuo, Sebastian Wurster, Ying Jiang, Jeffery Tarrand, Sung-Yeon Cho, Dimitrios P Kontoyiannis

**Affiliations:** UTH Houston/MD Anderson Cancer Center, Houston, Texas; The University of Texas MD Anderson Cancer Center, Houston, TX; The University of Texas MD Anderson Cancer Center, Houston, TX; The University of Texas MD Anderson Cancer Center, Houston, TX; University of Texas MD Anderson Cancer Center, Houston, TX, Houston, Texas; The University of Texas, MD Anderson Cancer Center, Houston, Texas; The University of Texas MD Anderson Cancer Center, Houston, TX

## Abstract

**Background:**

Historically cancer patients (pts) are considered to be at an increased risk of *Listeria monocytogenes* infection (listeriosis, LT), especially those with ongoing chemotherapy or receipt of glucocorticoids (GCs). However, the features of this uncommon infection have not been described in contemporary cohorts of cancer pts.

**Figure:**
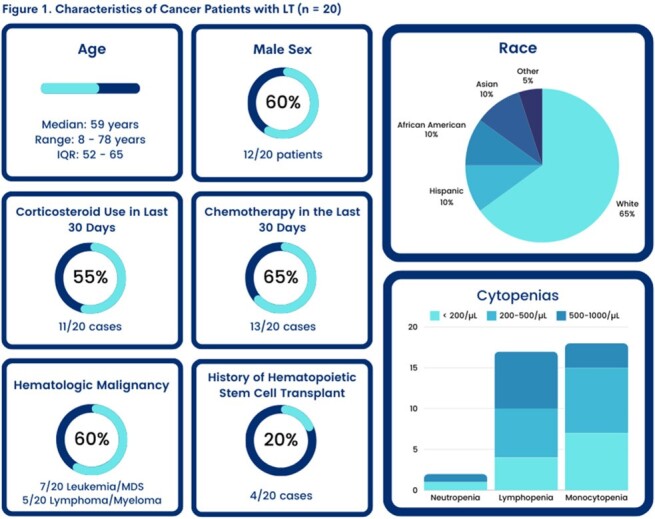

**Methods:**

We performed a 12-year retrospective chart review of cancer pts with LT as confirmed by positive culture from blood or other sterile clinical samples at MD Anderson Cancer Center (2011-2023).

**Figure:**
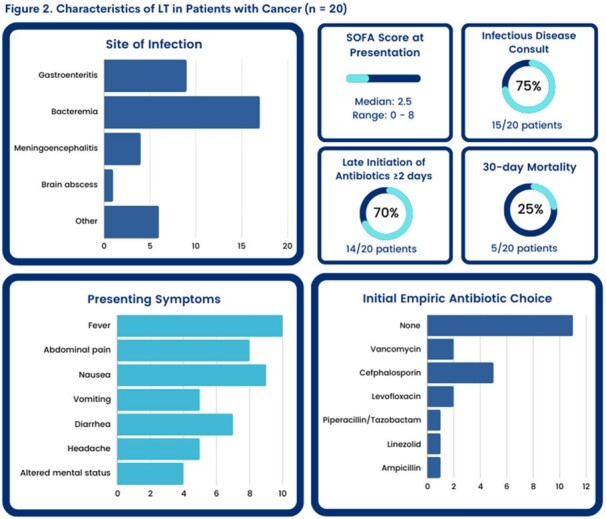

**Results:**

We identified 20 cancer pts with LT. Twelve (60%) had an underlying hematologic malignancy (Figure 1). Lymphopenia < 1000/µL (17, 85%, severe < 200/µL in 20%) and monocytopenia < 1000/µL (18, 90%, severe < 200/µL in 35%) were common at diagnosis, as well as chemotherapy (13, 65%) and GC use (11, 55%) within 30 days prior to LT diagnosis. Most (15 pts) were < 65 years old. Fever was seen in 10 pts. Vomiting/diarrhea were common (50%), while 45% of pts had headache or altered mental status. LT most often presented with bacteremia (17, 85%) and gastroenteritis (9, 45%) (Figure 2). 7/9 pts with gastroenteritis were bacteremic. Central nervous system (CNS) manifestations (meningitis, brain abscess) were documented in 2 pts who had a lumbar puncture. Focal non-CNS infections were seen in 30% of pts in unusual sites: cellulitis, abscess, endocarditis, cholangitis, urinary tract infection, and peritonitis. Of interest, 11pts (55%) had low SOFA score at presentation and were given antibiotics only after blood cultures were positive for *Listeria*. In fact, most pts (17, 85%) either did not receive empiric (n=11) or had inappropriate empiric antibiotics (n=6) until LT diagnosis. The 30-day mortality from LT diagnosis was 25% and correlated with high SOFA score ≥ 5 (5/8 pts, P = 0.0005). Delayed appropriate antibiotics were not significantly associated with increased 30-day mortality in this small cohort (Table 1).

**Figure:**
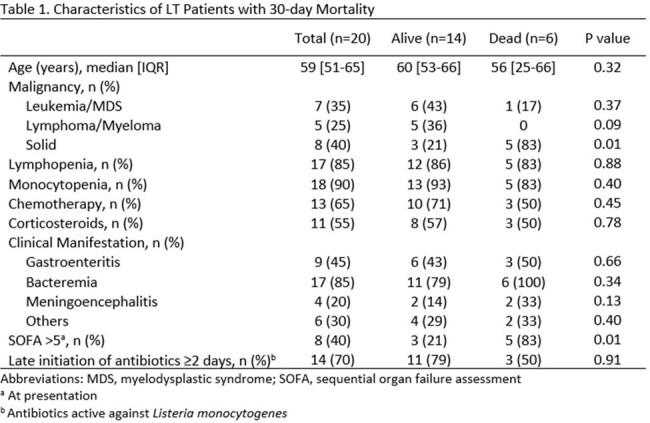

**Conclusion:**

Although uncommon, LT in cancer pts has pleotropic manifestations and variable morbidity, from non-bacteremic gastroenteritis to septicemia, CNS and non-CNS focal infections. Consider LT in ill cancer pts with lymphopenia/monocytopenia and recent chemotherapy or GCs, especially if they have GI symptoms, even in the absence of fever or CNS involvement.

**Disclosures:**

Sebastian Wurster, MD, MSc, Astellas Pharma: Grant/Research Support|Gilead Sciences: Grant/Research Support Dimitrios P. Kontoyiannis, MD, AbbVie: Advisor/Consultant|Astellas Pharma: Advisor/Consultant|Astellas Pharma: Grant/Research Support|Astellas Pharma: Honoraria|Cidara Therapeutics: Advisor/Consultant|Gilead Sciences: Advisor/Consultant|Gilead Sciences: Grant/Research Support|Gilead Sciences: Honoraria|Knight: Advisor/Consultant|Merck: Advisor/Consultant|Scynexis: Advisor/Consultant

